# Health workers’ adherence to the malaria test, treat and track strategy during the COVID-19 pandemic in malaria high transmission area in Eastern Uganda

**DOI:** 10.1186/s12936-023-04786-x

**Published:** 2023-11-27

**Authors:** Richard Kabaka Mumali, Charles Okolimong, Tonny Kabuuka, Yovani Moses Lubaale, Ambrose Okibure, Francis Okello, David Soita, Peter Olupot-Olupot

**Affiliations:** 1 Department of Community and Public Health, Busitema University Faculty of Health Sciences, Mbale, Uganda; 2 Mbale Clinical Research Institute, P.O. Box 1966, Mbale, Uganda; 3 National Agricultural Research Organization (NARO), National Livestock Resources Research Institute (NaLIRRI), P.O. Box 5704, Kampala, Uganda; 4 Varimetrics Group Limited, Mbale, Uganda

**Keywords:** Knowledge, Practices, Skills, Malaria diagnostic test, Anti-malarial, Malaria test, Treat, Track strategy

## Abstract

**Background:**

Coronavirus disease 2019 (COVID-19) pandemic affected malaria control activities in sub-Saharan Africa (SSA) resulting in 690,000 excess deaths in the year 2021. The authors hypothesized that COVID-19 affected the World Health Organization (WHO) Test, Treat and Track (T3) strategy that has been implemented in Uganda since 2010. In this study, health worker’s adherence to the T3 strategy during COVID-19 pandemic in Eastern Uganda was studied by assessing their knowledge, skills and practices.

**Methods:**

A cross-sectional study utilizing mixed quantitative and qualitative data collections methods was conducted at Mbale Regional Referral Hospital in Eastern Uganda between November and December in 2020. Data were captured on demographics, knowledge, skills and practices for both health workers (HWs) and patients. Quantitative data were analysed using STATA 15.0 and reported as descriptive statistics, proportions and statistical associations. Moreover, qualitative data were collected via key informant interviews (KII) among purposively sampled study participants and analysed thematically using NVIVO software. Ethical approval was obtained prior to the study.

**Results:**

A total of 436 study participants, of whom 103/436 (24%) and 333/436 (76%) were HWs and patients, respectively were studied. Among the HWs with mean age of 34 years (SD = 8.8 years), 81/103 (79%) had good practices, most 63/103 (61%) had good knowledge, and only 11/103 (10.7%) had good skills. Specifically, on the cadres, the laboratory personnel 19/103 (18%) had good knowledge 14/19 (74%) OR: 2.0 (95% CI 0.7–6) and were highly skilled OR: 4.6 (95% CI 1.2—18.1; *P* < 0.0150) compared to other cadres, respectively. Among the patients whose age ranged 3 months to 80 years (mean 17.8 years) and females 177/333 (53%); a majority 257/333 (77%) were tested, of whom 139/333 (42%) tested positive. Out of the positive cases, 115/333 (35%) were treated and tracked. About 75/333 (23%) were not tested but treated for malaria. Of the 168/239 (70.3%) patients tested, 115/168 (68.5%) were positive and treated, *P* = 0.0001. The KII revealed low level of In-service training, overwhelming number of patients and stock-out of supplies as a key factor for poor HW adherence to T3 strategy.

**Conclusions:**

During COVID-19 pandemic period HWs adherence to T3 initiative was low as 27% malaria patients did not receive treatment.

## Background

Globally, malaria remains a public health problem. The global tally of malaria cases reached 247 million in 2021 compared to 245 million in 2020 and 232 million in 2019; corresponding to a continued rise of malaria cases between 2020 and 2021 [1, 2]. Sub-Saharan Africa (SSA) contributes to approximately 80% of global disease burden. In Uganda, hospital records suggest that malaria is responsible for 30 to 50% of outpatient visits (OPD) visits, 15 to 20% of admissions, and 9 to 14% of inpatient (IP) deaths [3]. Uganda ranks third in the total number of infections after the Democratic Republic of the Congo (DRC) and Nigeria [4, 5]. Recently malaria control has been grossly affected by COVID-19 pandemic with detrimental effect on prevention, treatment and outcomes [6–9]. As part of the response to the pandemic, the Uganda government, like many others, enforced several lockdowns (LDs) over a period of 2 years (2020 – 2021). Subsequently, these LDs negatively impacted on healthcare services in SSA especially those for long standing endemic diseases including malaria, HIV and Tuberculosis (TB) [4–6]. The COVID-19 ramifications also affected national economies in the sub-continent [6, 7]. Emerging data reveal that LDs did more harm than good [12]. For instance, malaria control activities were most affected during the LDs which stagnated or tended to reverse efforts on renewed interest on the disease elimination [6–8]. In 2010, the World Health Organization (WHO) rolled out an T3 strategy that shifted practice from presumptive treatment of fever with anti-malarial to evidence based targeted test, treat and track for malaria (T3 Initiative), subsequently, the Uganda Ministry of Health (MOH) adopted these guidelines [13]. This period also corresponded to the wide spread use of malaria rapid diagnostic tests (RDTs) [14] and artemisinin-based combination therapy (ACT) for malaria treatment. In line with the T3 strategy, Uganda adopted malaria parasitological diagnosis and prompt treatment with ACTs as a measure for reducing morbidity and mortality from the disease [10, 11]. Elsewhere the T3 strategy efforts have faced some challenges arising from inadequacies on systems and practices [12, 13]. The practice of presumptive treatment of malaria continues to persist in many settings in SSA. Although the proportion of malaria cases being tested in SSA has increased across the sub-continent, performance of the T3 strategy remains suboptimal. For instance, in Uganda, testing for malaria increased from 39% in 2018/2019 to 77% in 2020 [14, 15]. Similarly, a cross sectional study carried out in Mfantseman municipality in the Central Region of Ghana indicated that the proportion of OPD malaria cases that were tested increased from 39% in 2013 to 78% in 2016 [15, 16]. Presumptive prescriptions and treatment to persons with negative tests in SSA has remained highly prevalent [17–19]. Attendant consequences for presumptive treatment include frequent stock-out of diagnostic test kits, reagents and anti-malarials [14, 20, 21]. These are compounded when HWs do not adhere to the T3 strategy. The ramifications of non-adherence to the T3 strategy for malaria include incorrect treatment and delays in treating malaria or other causes of fever. This can however be over come through strengthening the implementation of the T3 strategy [11, 15]. Based on MOH guidelines in Uganda, adherence to the T3 strategy is key to controlling and subsequently eliminating malaria in the country [14, 15, 22].

In this study, the T3 strategy is described with the aim of determining HWs adherence to the malaria test, treat and track strategy during the COVID-19 pandemic. Moreover, whether HW’s knowledge, skills and practices affected adherence to the T3 strategy in Mbale Regional Referral Hospital (Mbale RRH) in Eastern Uganda was studied.

## Methods

This was a cross-sectional study conducted between November and December 2020 at Mbale Regional Referral Hospital (MRRH) in Eastern Uganda; an areas recently profiled for unusual clinical spectrum of severe malaria [5]. By the time of this study, the region had experienced two waves of COVID-19 as was the rest of the country. A structured questionnaire for quantitative data collection and key informant interviews for qualitative data collection were used to assess knowledge, practices and skills of health care providers on T3 strategy, while observational checklist and review of records were employed to obtain data on the T3 strategy.

### Study population

The study population included randomly selected 103 practicing health workers and 333 patients who visited Mbale Regional Referral Hospital (Mbale RRH). In addition, 06 key informants were purposively studied strictly for qualitative aspects. Only patients with suspected malaria and respondents at the study area at the time of study who consented were eligible for inclusion. Patients not suspected of malaria or were too sick to respond and Health workers who did not consent or absent to participate in T3 strategy were excluded.

### Sampling and sample size determination

The Kish-Leslie’s formula: **n**=$\frac{{z}^{2}\alpha /2pq}{{\delta }^{2}}$ of 1965 for descriptive studies was used to estimate the minimum sample size for health workers and client-exit interviews with a 10% non-response rate resulting into a sample size of 428 study participants [23] Where; n = study sample size required, Z is the z score corresponding to the chosen alpha level of 0.5 (95% confident interval) which is 1.96, P = 50% estimated proportion to mean at least 50% of febrile patients at MRRH conforming to the T3 strategy which is 50% from previous study [23], q is 1-p and δ is the estimated margin of error—Akos Odikro M et al. recommended using 0.05 (5%) [24]. Substituting values into the equation, the sample size was estimated as $({1.96}^{2}$*$0.5 {(1-0.5)}^{2}/{0.05}^{2}$= 385. A 10% non-response rate has also been considered, hence 428 participants where targeted for this study. Both probability and non-probability sampling techniques were used. Simple random sampling was used to select participants for exit interviews because of the small population, homogeneous & readily available. Subjects in the population were sampled by a simple random process without replacement, using numbers (P) for participation and (NP) not participating written on piece papers folded, mixed in a tin, powered in a basin and picked at random by consented/ assented participants. In so doing each person remaining in the population had the same chance of being selected for the sample. The total of 436 study participants took part in the study, a minimum of 20 participants were interviewed daily for 2 months to obtain the required data. While purposive sampling was used to select 6 key informants for the key informant interviews.

### Data collection

Data were collected from Nov to Dec 2020, from three main sources: patients, HWs and review of records. Data were collected on demographic characteristics and knowledge of HWs on T3 strategy. While observation checklist was used to collect available data on practices and skills of HWs in malaria T3 strategy. The data regarding the proportion of patients tested, treated and tracked among those screened was obtained through records reviewed and observational checklist. The outcome variable, adherence to the T3 strategy, is a composite variable that combines testing, treating and tracking of malaria. It was a derivative of three components of the strategy that is Testing + Treating + Tracking = T3 adherence. To meet the criteria for the dependent variable, the health worker should have requested for RDT, Blood slide or both tests for all malaria suspects, treated confirmed malaria cases with anti-malarials and asked the patient to return for follow-up. If all these were done, it implied health worker adhered to the malaria T3 strategy.

### Data analysis

Data-entry was done using Microsoft excel 2013. The quantitative data was then exported to STATA 15.0 for analysis into uni-variate and bi-variate analysis. Summary descriptive statistics were conducted and presented as frequencies and proportions, in tables. Univariate analysis was performed to determine the crude association between outcome variables and other predictor variables using odds ratios and 95% confidence intervals (CIs). A p-value of less than 0.05 was considered significant. A bivariate analysis was conducted using chi-square distribution to determine the association between health worker’s factors associated with T3 strategy. In determining a combination of health worker’s factors associated with adherence to T3 strategy, the outcome variables and all the exposure variables that predicted the outcome p < 0.1 in the crude analysis were placed in a multiple logistic regression model. These variables included demographic characteristics, knowledge, skill, and practices of HWS in T3 strategy. Associations were considered significant at P-Value of 0.05 or less. Qualitative data collected using key informed interviews were audio recorded while generating the notes. The key informant guide consisted of questions designed to assess and document the impact of Covid 19 on T3 strategy. This was analysed using thematic content with the help of NVIVO software.

### Ethical approval and consent to participate.

Approval for this study was obtained from Uganda National Council of Science and Technology and MRRH research and ethics committee (MRRH-REC), with approval no MRRH REC-OUT-011–2020. Formal permission was also sought from the director MRRH. Written consent from respondents was obtained before questionnaires were administered. Confidentiality and privacy of patient documents and information was maintained throughout and thereafter.

## Results

A total of 436 study participants of whom 103/436 (24%) and 333/436 (76%) were HWs and malaria suspects; respectively were studied. Among the HWs, the mean age was 34 years (SD = 8.8 years), 57/103 (55%) were females and 54/103 (52%) had attained tertiary education. On T3 strategy, 81/103 (79%) had good practices. Most 63/103 (61%) had good knowledge based on assessment of five questions, all carrying equal score, where 78/103 (76%) knew definition of RDTs, 57/103 (55%) knew turnaround time (TAT) for RDTs, majority 72/103 (70%) knew it was very important to test malaria suspects before treatment. Only 11/103 (10.7%) had good skills as 7/103 (7%) of HWs knew correct procedures of performing RDT, 16/103 (16%) would correctly perform blood slide (BS), less than half 45/103 (44%) knew how to report results for BS. Among the laboratory personnel 14/19 (74%) had good knowledge, were 2.0 times more likely to be knowledgeable compared to other cadres OR: 2.0 (95% CI 0.7–6). In addition, they were 4.6 times more likely to be highly skilled compared to other cadres (OR: 4.6; 95% CI 1.2–18.1; *P* < 0.0150) (Table 1).

**Table 1:** Association between level of knowledge, skills, and practices of health workers in malaria management (T3) and demographic characteristics

Variable	Freq n = 103 (%)	Knowledge level			Skills			Practices of health workers on patients			
		Poor	Good	COR (95% CI)	Unskilled	Skilled	COR (95% CI)	All sample n = 333 (%)	Poor	Good	COR (95% CI)
Sex											
Male	46 (45)	13 (28)	33 (72)	2.3 (1.0, 5.3)**	40 (43.5)	6 (54.5)	1.6 (0.4,5.5)	156 (47)	33 (21)	123 (79)	1.0 (0.6, 1.6)
Female	57 (55)	27 (47)	30 (53)	0.4 (0.2, 1.0)**	52 (56.5)	5 (45.5)	0.6 (0.2,2.3)	177 (53)	36 (20)	141 (80)	1.1 (0.6, 1.8)
Age-group											
< 30	35 (34)	16 (46)	19 (54)	0.6 (0.3, 1.5)	32 (34.8)	3 (27.3)	0.7 (0.2,2.9)	256 (77)	58 (23)	198 (77)	0.6 (0.3, 1.2)
30–39	40 (39)	11 (28)	29 (73)	2.2 (0.9, 5.4)	34 (37.0)	6 (54.5)	2.0 (0.6,7.3)	29 (9)	2 (7)	27 (93)	3.8 (0.9, 16.6)*
40–49	18 (18)	7 (39)	11 (61)	1.0 (0.3,2.8)	18 (19.6)	0 (0.0)	–	14 (4)	0 (0)	14 (100)	–
50 +	10 (10)	6 (60)	4 (40)	0.4 (0.1,1.5)	8 (8.7)	2 (18.2)	2.3 (0.4,12.9)	34 (10)	9 (27)	25 (74)	0.7 (0.3, 1.6)
Level of education											
Secondary	2 (2)	1 (50)	1 (50)	0.6 (0.03, 10.5)	2 (2.2)	0 (0.0)	–	52 (16)	5 (10)	47 (90)	2.8 (1.0, 7.3)
Tertiary institution	54 (52)	21 (39)	33 (61)	1.0 (0.4,2.2)	47 (51.1)	7 (63.6)	1.7 (0.5,6.2)	15 (5)	4 (27)	11 (73)	0.7 (0.2, 2.3)
University	47 (46)	18 (38)	29 (62)	1.0 (0.4,2.3)	43 (46.7)	4 (36.4)	0.7 (0.2,2.4)	4 (1)	0 (0)	4 (100)	–
Cadre											
Lab personnel	19 (18)	5 (26)	14 (74)	2.0 (0.7,6.1)	14 (15.2)	5 (45.5)	4.6 (1.2 18.1)**				
Clinician	16 (16)	5 (31)	11 (69)	1.5 (0.5,4.7)**	15 (16.3)	1 (9.1)	0.51 (0.1, 4.4)				
Nurse	42 (41)	21 (50)	21 (50)	0.5 (0.2,1.0)	39 (42.4)	3 (27.3)	0.5 (0.1, 2.1)				
Medical Officer	17 (17)	9 (53)	8 (47)	0.5 (0.2,1.5)	16 (17.4)	1 (9.1)	0.5 (0.1, 4.0)				

^**^P < 0.05, *p < 0.01

**Fig. 1:**
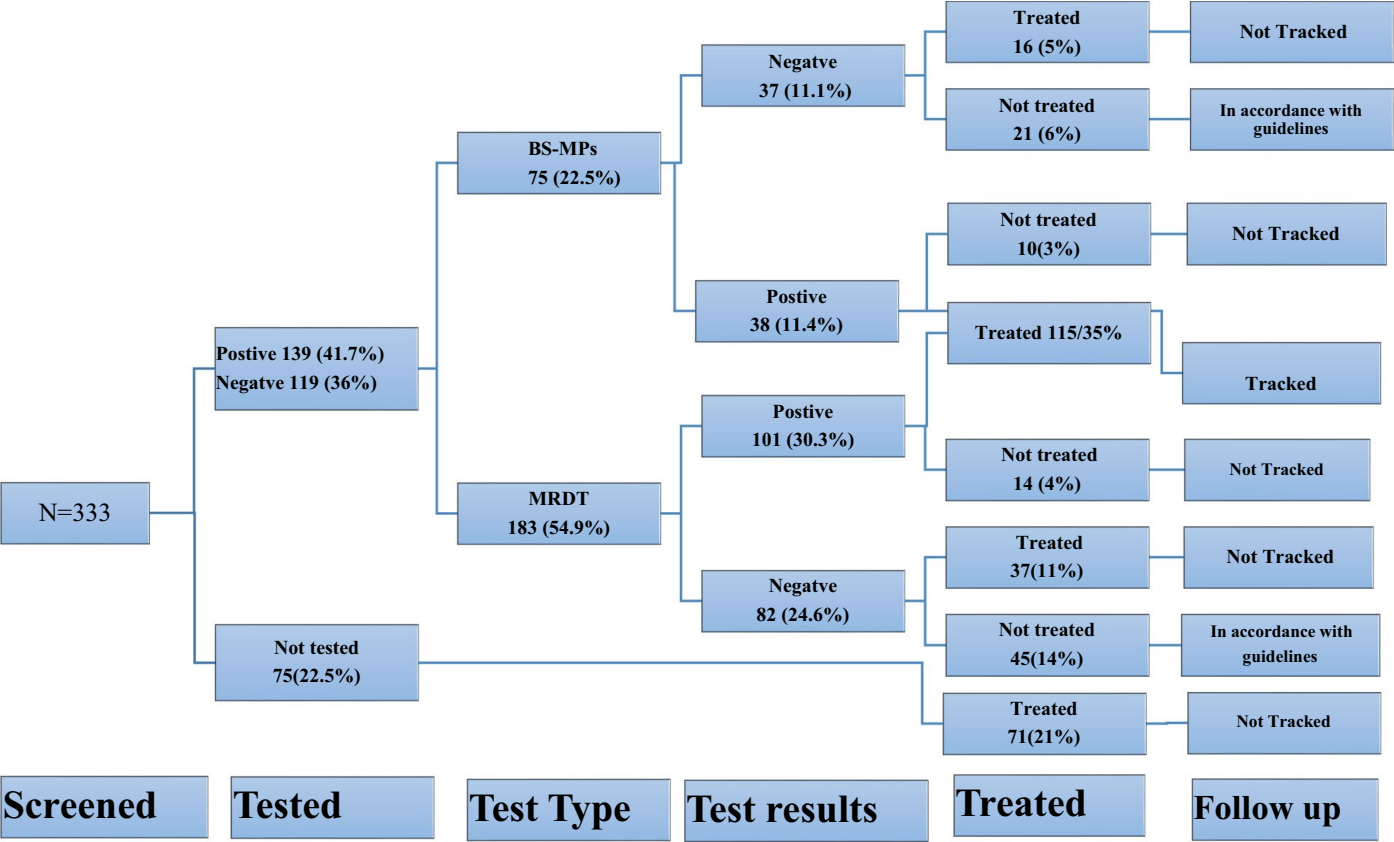
Flow chart for determining malaria test, treat, and track strategy among malaria suspects

Among suspected malaria patients screened, 177/333 (53%) were females, the majority 256/333 (77%) were aged between 3 months and 80 years, and the mean age was 17.8 years, (SD ± 18.5 years). Less than half 132/333 (40%) had no formal education mainly because of age (< 5 years). Overall majority 264/333 (79%) of malaria suspects reported health worker’s good practices of malaria diagnosis and treatment in MRRH, 249/333 (75%) of malaria suspects returned laboratory test results to clinicians, majority 331/333 (99%) who visited the pharmacy was opened during working hours, most 258/333 (78%) had blood drawn for malaria RDT or BS test, 249/333 (75%) received malaria test results after diagnosis in the laboratory. Similarly, most 237/333 (71%) of febrile patients referred to the laboratory for malaria laboratory tests, 256/333 (77%) were tested, less than a half 140/333 (42%) tested positive. Of the 239/333 (72%) patients who were tested and treated in MRRH, confirmed cases 115/333 (35%) received prescribed recommended ACTs and tracked or followed-up for review between 5 and 7 days. However, 66/333 (20%) of malaria suspects tested negative for both BS and RDT were neither treated nor tracked which is in accordance with National treatment guidelines, Fig. 1. Flow chart for determining malaria test, treat, and track strategy among malaria suspects. Overall, the proportion of febrile patients who were managed according to the T3 strategy for malaria control was 35%. Majority 168/239 (70.3%) of the patients who received treatment had been tested, but not all of them were positive for malaria since 115/168 (68.5%) tested positive, *P* = 0.0001 (Fig. 1).

### Qualitative data

Concerning testing and treating, while most key informants (KI) reported that within last 6 months they have not or occasionally run out of stock for RDT, they also agreed that other test method (malaria microscopy) would be available in any case.

*“We always have test kits and reagents in the laboratory”. KI-004*

*Rapid Diagnostic Tests (RDTs) and Reagents – not sure of the stock-outs because I haven’t followed up but at least one has to be available when patients are asked to do a test. KI-002*

Through review of stock cards, it was observed that there were no stock-outs of anti-malarials during the study period. However, according to qualitative data collected via key informant interviews (KII) from six key informants on availability of anti-malarials and associated factors to stock-outs. They reported occasional stock-outs of anti-malarial drugs and factors contributing to stock-outs of anti-malarials in MRRH before and during Covid 19 pandemic.

*“The hospital serves bigger region (Catchment area) 16 district under MRRH, so supplies gotten from National Medical Stores (NMS) may not be enough all the time in in tandem with patient’s numbers”. KI-001*

*“Overwhelming numbers of patients in the hospital. We are covering 16 districts and most referrals are self-referral (people come here for medicine by themselves), it being a referral hospital” KI-002*

*“This can be linked to misuse of drugs, irrational prescription of drugs to patients without confirming that they have malaria and high malaria cases at that particular time”. KI-005*

*“Clinicians prescribe drugs at the same time with test request to the laboratory in the books. (patients get medicine before testing /going to the lab) and some people pick drugs for their friends and relatives without laboratory results”. KI-006*

About T3 strategy continual professional development opportunities, respondents said since Covid-19 outbreak, they have not yet had any more trainings as a hospital, no virtual seminars held yet because of interruption by COVID -19 pandemic and its negative impact.

*“Yes, continuous medical education (CME) and trainings for prescribers used to be carried out regularly unfortunately because of Covid-19 pandemic it has halted most of the trainings and CMES. We have not had any training so far since outbreak of covid-19 pandemic”. KI- 002*.

## Discussion

This study sought to determine the level of HWs’ adherence to T3 strategy and its associated factors during COVID-19 pandemic in Eastern Uganda. The study findings revealed that only (43.3/103) 42% of the HWs and (115/333) 35% of the patients adhered to the T3 strategy. The noncompliance by health workers to test all malaria suspects prior to administration of anti-malarials and, failure to review or follow-up of patients on treatment were significant factors in this study. Elsewhere, some data have been published on the T3 strategy, however, these data need updating because they were done in early stages of initiation of the strategy [11, 15, 24]. Moreover, the picture remains incomplete because other settings have not contributed data on the same. For instance, in Eastern Uganda, despite being malaria perennial high transmission area, have no formally published data on the T3 strategy. Scarcity of these data potentially affects evidence-based disease control. Therefore, findings in this study could inform best practices. For example, factors that were associated with the adherence to the T3 strategy included knowledge, practices, skills and social demographic characteristics could be harnessed for improvement of disease control through this strategy. Furthermore, use of these data could include correlation of T3 strategy with patient outcome [11–14], and identification of gaps that require addressing.

This study observed that a majority 258/333 (78%) of suspected malaria case patients were either tested by Rapid diagnostic test (RDT) 183/333 (54.9%) or Blood slide (BS) 75/333 (22.5%). Studies elsewhere within SSA have reported varying, but consistently lower testing rates of suspected malaria cases ranging from 43.5 to 64.6% [24–26]. This variation in testing rate could be attributed to the differences in the settings and availability of testing services. During the COVID-19 pandemic, it was observed that clinical diagnosis using fever as a prognosticator of malaria was widely used mainly because of erratic supplies of testing kits, laboratory supplies and or health personnel affected by LDs in Eastern Uganda. Using clinical diagnosis may have resulted in over diagnosis of malaria and poor adherence to T3 strategy [11, 16, 24]. Moreover, this could have also caused stock-outs of anti-malarials and /or underutilization of testing kits and supplies. This study now shows that up to 27% of patients missed anti-malarial drugs, thereby affecting the pre-covid era treatment target of 78% [6–8]. This low level of T3 adherence can only be attributed to the effects of COVID-19 and an enormous strain on the health care delivery system as have been recently described [9, 27]. Whereas data validating accuracy of clinical diagnosis for malaria is old [25], recent emergence of RDT negative malaria infection due to HRP-2 deletion [28, 29], calls for re-evaluation of clinical criteria in settings where accurate blood slide results may be difficult to obtain [30]. For future epidemics and pandemics where low adherence to malaria T3 strategy is likely to be repeated, two options may be considered. Early preparations and having alternatives are important. For only the duration of the pandemic, clinical diagnosis could be adopted and reported as T3c (c = clinical) that enable patients access treatment on clinical basis in a bid to improve patient outcomes. Alternatively, the T3 strategy could be downgraded to ST2p (Treat and Track suspected case of malaria during the pandemic). Elsewhere, geographical variations in adherence to T3 strategy have been reported. Studies have shown that in rural settings, testing rates were higher compared to urban settings [24, 31]. There are contemplations that geographical location alone was not the factor behind these high rates of testing for malaria in rural settings. Instead, this could be because in such settings RDTs are mainly used for lack of equipped laboratories. Moreover, RDTs takes less turnaround time (TAT) of 15–20 min compared to the laborious and time-consuming microscopy. The findings indicate few 22.5% BS-MPS compared to 55% RDTs. This difference may be attributed to inadequate health worker’s skill, geographical locations, type of test done and time of the study [32, 33]. This study reports that during the COVID-19 pandemic, febrile patients were prescribed anti-malarials irrespective of whether they were tested or not. Clinicians prescribed ACT for majority 70.3% of the patients tested.

Adherence to malarial national treatment guidelines (MNTGs) is critical for disease control. However, these have varied from one setting or country to another. For instance, data have shown varying rates of 44% in Nigeria to 67.1% in Malawi [10, 11, 34]. The varying percentages of clinician adherence to recommended MNTGs can possibly be attributed to the availability of the testing facilities and recommended anti-malarials in health facilities. Clinicians in this study were more likely to prescribe treatment based on their knowledge and practices without testing or utilizing test results. This could be attributed to high level of knowledge and some experiences that has been obtained over time when all fevers were presumed to be caused by malaria and possibly lack of understanding to adhere to T3 strategy [20, 35, 36].

Tracking of patients treated for malaria is essential in determining treatment outcomes and informing progress in disease elimination [37]. This study indicated that less than 50% of the patients treated were tracked and similar to trends in Bongo and Ho districts of Ghana with 30.7% [31, 38]. However, in studies done by Kankpetinge et al*.* and by Mubi et al., higher percentage of tracking of patients was realized at Atebubu-Amanten district, Ghana, where over 90% of all patients treated for malaria were reviewed [11, 24]. It remains to be validated whether tracking directly contributes to the disease control, but from the clinical point of view, it is useful for documenting patients’ outcomes including survival and complications.

Overall findings from this study indicated that at MRRH during the COVID-19 pandemic, about one third of malaria patients 115/333 (35%) were treated according to T3 strategy as opposed to the target of 70.3%. The low T3 rates were mainly due to the ramifications of COVID-19 on the health systems [8]. The rates reported in this study were even lower than the rates in 2006 in the Bongo district in Ghana at 42.5% [13, 15].

The study revealed 61% good knowledge level on malaria T3 strategy. This is slightly lower compared to a study conducted by Prah et al*.* among Ghanaian prescribers assessing the knowledge, attitude and practices (KAP) regarding malaria diagnosis, which revealed a good knowledge level 73% of respondents, this could be due intense training and capacity building provided to them [12, 38, 39]. However, the previous study identified several barriers to the test-based management of malaria reported by Ghanaian prescribers, that includes reliance of strong clinical suspicion of malaria in patients, mistrust in parasitological tests and increasing workload at the clinics which is consistent with this study.

More than half 55% of patients were tested using RDT compared to 22.5%. A study done by Kabaghe et al*.* [40] established that, most HWs were happy to use RDTs compared to microscopy, because they are fast and can easily be used when handling many patients. This is in agreement with the study carried out in Uganda by Talisuna et al*.* [41], about the changing landscape of malaria case management following the policy change, the proportion of tests by RDT increased to about 55% compared to 30% by microscopy [24, 25, 29].

### Study limitations and strength

This study had limitations in that there was no study for pre and post COVID-19 levels of adherence to T3 in our settings for comparison on trends, however, given the fact that the targets for T3 in the pre COVID-19 era were already set, our findings underpin the effect of COVID-19 on malaria T3 control strategy. Health workers were interviewed at their workplace, well aware of the interview biases, data completeness and accuracy, mitigated by training and continuous supervision of research assistants in the field to ensure quality and consistency of the collected data. Nevertheless, the strength of this study rests in the rigorous mixed quantitative and qualitative methods and validation of findings from secondary data using interviews to assess health worker’s adherence to the T3 strategy for malaria control in Eastern Uganda.

## Conclusion

During COVID-19 pandemic, HW’s adherence to malaria T3 was below expected levels of 50% in Eastern Uganda. About 27% confirmed malaria patients did not receive treatment. Inadequate skills were limiting factor to the success of the T3 strategy. High levels of knowledge and practices were found to be adequate. More of malaria microscopy as a gold standard should be performed to improve T3 adherence. Deployment of alternative approaches such as T3c and ST2p to improve on malaria patient outcomes should be considered during disease outbreaks, epidemics or pandemics. More research on T3 strategy in different settings is recommended.

## Data Availability

The datasets used and/or analysed during the current study are available from the corresponding author on reasonable request.

## Abbreviations

ACT: Artemisinin-Based Combination Therapy
BS: Blood Smear or slide stained with field stains and examined by microscopy
CMES: Continuous medical education
COVID-19: Coronavirus disease
KI: Key informants
MRRH: Mbale regional referral hospital
NTGs: Recommended national treatment guidelines
RDT: Rapid diagnostic test
REC: Research and ethics committee
T3: Malaria test, treat and track strategy
TAT: Turnaround time
WHO: World Health Organization
